# Bovine Astrovirus—A Comprehensive Review

**DOI:** 10.3390/v14061217

**Published:** 2022-06-02

**Authors:** Qinghe Zhu, Bin Li, Dongbo Sun

**Affiliations:** 1 Heilongjiang Provincial Key Laboratory of the Prevention and Control of Bovine Diseases, College of Animal Science and Veterinary Medicine, Heilongjiang Bayi Agricultural University, No. 5 Xinfeng Road, Sartu District, Daqing 163319, China; qinghezhumy@126.com; 2 Key Laboratory of Veterinary Biological Engineering and Technology, Ministry of Agriculture, Institute of Veterinary Medicine, Jiangsu Academy of Agricultural Sciences Nanjing 210014, China

**Keywords:** bovine astrovirus, epidemiology, evolution, pathogenesis, potential cross species transmission

## Abstract

Bovine astrovirus (BoAstV) is a small non-enveloped virus with a single-stranded positive-sense RNA. In 1978, BoAstV was first found in calf diarrhea fecal samples in the United Kingdom and since then it has been reported in many other countries. It has wide tissue tropism and can infect multiple organs, including the intestine, nerves and respiratory tract. Since BoAstV is prevalent in healthy as well as clinically infected bovines, and is mostly associated with co-infection with other viruses, the pathogenic nature of BoAstV is still unclear. At present, there are no stable passage cell lines available for the study of BoAstV and animal model experiments have not been described. In addition, it has been reported that BoAstV may have the possibility of cross-species transmission. This review summarizes the current state of knowledge about BoAstV, including the epidemiology, evolution analysis, detection methods, pathogenesis and potential cross species transmission, to provide reference for further research of BoAstV.

## 1. Introduction

Astroviruses (AstV) belong to the *Astroviridae* family, including two genera *Mamastrovirus* (MAstV) which can cause diarrhea and neurological symptoms in mammals, and *Avastrovirus* (AAstV), which can lead to hepatitis, nephritis and diarrhea in birds [1]. Among MAstV, bovine astrovirus (BoAstV) was first reported in calf diarrhea stool samples in the United Kingdom in 1978 and subsequently in healthy and diarrheic cattle in many countries [2,3,4,5,6,7,8,9,10,11]. In recent years, it has been reported that BoAstV has been detected not only in intestinal system samples, but also in brain tissues of animals with neurological symptoms [2,12,13]. Evolutionary analyses showed that astrovirus strains from bovine brain tissue were closely related to astrovirus strains from humans, pigs, sheep and other animals with neurological symptoms, indicating that cross-species transmission may occur. Some studies have also proposed the potential pathogenic role of these viruses [2,6], but many questions remain unresolved. This review describes the current molecular epidemiology, genome structure, classification, detection methods, pathogenesis and potential cross species transmission of BoAstV.

## 2. Discovery and Epidemiology

In 1978, some six-pointed star pattern virus particles with a diameter of about 28 nm were detected in the fecal samples of British diarrheic calves by electron microscopy, and were named astroviruses. From limited experiments, it was concluded that these viruses could infect calves, but did not cause diarrhea or clinical illness [9]. Another study showed that the detection rate of BoAstV in healthy calves was higher than that in diarrheic calves, but the difference was not significant [8]. More recent studies showed that the overall prevalence of BoAstV in diarrheic and asymptomatic calves was 55.17% and 36.36%, respectively, indicating that there was a correlation between astrovirus infection and calf diarrhea [11].

**Figure 1:**
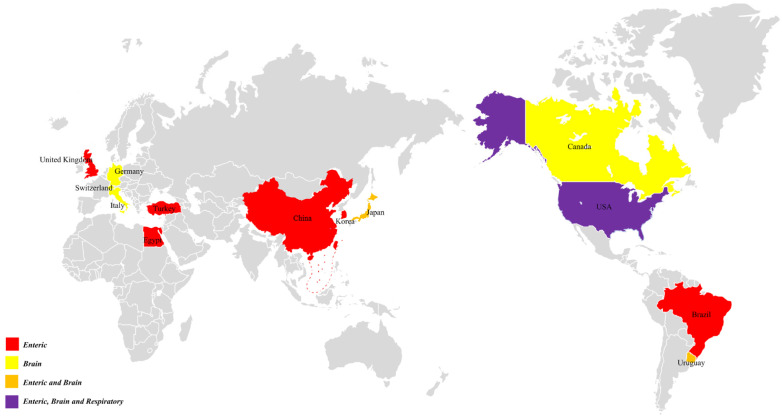
Major countries representing the identification of BoAstV.

Since 1978, the virus has been reported throughout the world, as seen in Figure 1. Although BoAstV has been confirmed in many countries, its prevalence may vary depending on geographical location, age and herd management (Table 1). The first report showed that 16 of 59 blood samples and 11 of 22 herds were serologically positive to the astrovirus-like agent in the United Kingdom using the fluorescent antibody test (FA) [9]. In 1985, 28 of 1060 fecal samples of diarrhea in three-day to three-week-old calves in the USA were positive for BoAstV, with a positivity rate of 2.64% [10]. In 2011, five of 209 adult bovine fecal samples in China were positive for astrovirus by reverse transcription polymerase chain reaction (RT-PCR), with a positivity rate of 2.39% [14]. In 2014, nine of 115 fecal samples were positive for BoAstV in Korea, with a positivity rate of 7.83% [6]. In 2015, 15 of 146 fecal samples were positive for BoAstV in Japan, with a positivity rate of 10.27% [15], 88 of 135 fecal samples were positive for BoAstV in the United Kingdom, with a positivity rate of 65.19% [8] and in China, 211 rectal swabs collected from cattle and buffalo calves with mild to severe diarrhea were tested for BoAstV by RT-PCR with a positivity rate of 43.6% [16]. In different Brazilian states, a prevalence of 14.34% of BoAstV in fecal samples from 272 head of cattle was detected [4]. In 2017, eight of 25 fecal samples were positive for BoAstV in Egypt, with a positivity rate of 32% [5]. In 2018, four of 127 fecal samples from diarrhea calves were positive for BoAstV in Turkey, with a positivity rate of 3.15% [17]. In 2019, 128 of 500 fecal samples from diarrheic calves were positive for BoAstV in Uruguay, with a positivity rate of 25.6% [18]. In 2021, fecal samples from 164 calves from 12 provinces in China were collected and BoAstV was detected with RT-PCR, with an overall prevalence of 46.34% [11].

In recent years, in addition to frequent detection in fecal samples, including the USA [19], Switzerland [2], Germany [20], Canada [21,22], Japan [23], Uruguay [24] and Italy [25], BoAstV was also detected in the brain tissue samples of cattle with neurological symptoms. The positivity rate in retrospective analysis can be as high as 85.71% [26]. In 2015, BoAstV was detected for the first time in four bovine nasal swab samples with respiratory symptoms, with a positivity rate of 8% [27]. There are some differences in the prevalence and histotropism of BoAstV in different countries and the continuous emergence of countries with positive astrovirus infection also shows that BoAstV is spreading all over the world. However, although the virus may likely have spread in Oceania and African countries, there are no reports of positive BoAstV cases in these regions. Further analysis also confirmed that the infection rate of young cattle is higher than that of older cattle, where BoAstV is common in 74% of calves, but not in 15% of adult cattle [6,8]. In calves, maternally-derived antibodies may play a significant role in the prevention of BoAstV infection, but this hypothesis needs further investigation.

**Table 1:** The transmission of BoAstV.

Sample Type	Year	Country	Positive Rate	References
blood samples	1978	United Kingdom	27.12% (16/59)	[9]
fecal samples	1985	USA	2.64% (28/1060)	[10]
	2011	China	2.39% (5/209)	[14]
	2014	Korea	7.83% (9/115)	[6]
	2015	Japan	10.27% (15/146)	[15]
		United Kingdom	65.19% (88/135)	[8]
		China	43.6% (92/211)	[16]
		Brazil	14.34% (39/272)	[4]
	2017	Egypt	32% (8/25)	[5]
	2018	Turkey	3.15% (4/127)	[17]
	2019	Uruguay	25.6% (128/500)	[18]
	2021	China	46.34% (76/164)	[11]
brain tissue samples	2013	USA	12.5% (4/32)	[19]
	2014	Switzerland	22.73% (5/22)	[2]
		Germany	-	[20]
	2016	Switzerland	85.71% (12/14)	[26]
		Switzerland	34.02% (33/97)	[7]
		Canada	44.44% (4/9)	[21,22]
		Japan	0.67% (1/150)	[23]
	2017	Switzerland	0.11% (2/1816)	[12]
	2018	Uruguay	2.7% (1/37)	[24]
	2019	Italy	0.36% (1/280)	[25]
nasal swab samples	2015	USA	8% (4/50)	[27]

## 3. Genomic Characteristics

**Figure 2:**
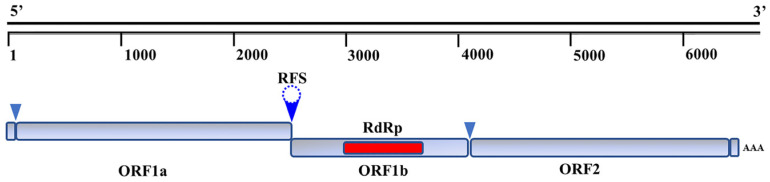
The genome structure of BoAstV. The genome of BoAstV contains three ORF regions that encode three proteins. The sequences for ORF1a and ORF1b overlap in the genome.

The BoAstV is a small, non-enveloped virus with a non-segmented, positive sense, single-stranded RNA genome of 6.2 to 7.7 Kb. The genome structure of BoAstV, which is similar to that of other known astroviruses, includes a 5′-untranslated region (UTR), three sequential open reading frames (ORFs) (ORF1a, ORF1b, and ORF2), 3′-UTR, and poly(A) tail [2], as seen in Figure 2. Of these, ORF1a encodes a 90 kDa non-structural protein nsp1a, that contains a conserved serine-like protease motif. ORF1a and ORF1b overlap each other, and the overlapping region between ORF1a and ORF1b contains the highly conserved “slippery heptamer” sequence 5′-AAAAAAC-3′. The ORF1b encodes an RNA dependent RNA polymerase (RdRP) [14].

The ORF2 encodes a capsid protein, which contains an N-terminal capsid core and a C-terminal spike domain. Studies have shown that the spike domain induces neutralizing antibodies [28]. In addition, the amino acid (aa) sequence of the ORF2 region is used as the basis for the classification of AstV. AstVs with aa sequence homology of the ORF2 protein greater than 75% are classified as the same species [29].

## 4. Phylogenetics and Evolution

Astroviruses are classified within the *Astroviridae* family, which was initially comprised of a single genus, *Astrovirus*, based on virion morphology [30]. The international committee on the taxonomy of viruses (ICTV) confirmed the classification of the *Astrovirus* genus within the family *Astroviridae* in 1995. In 2004, two genera were identified, known as *Mamastrovirus* (MAstV) and *Avastrovirus* (AAstV), which infect mammalian and avian species, respectively [31]. According to the current classification system, BoAstV belongs to the *Astroviridae* family and *Mamastrovirus* genus, which have been detected in intestinal, brain and respiratory samples [2,9,27].

**Figure 3:**
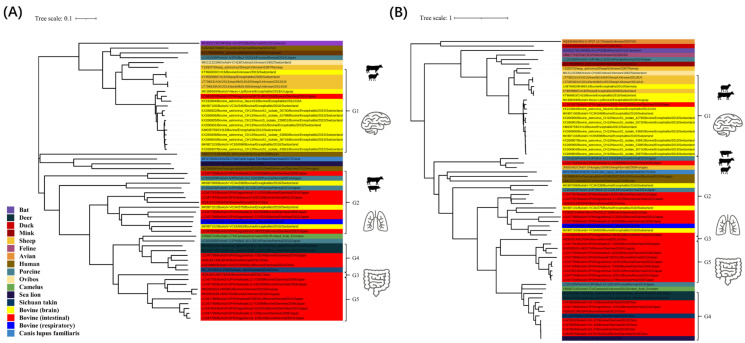
Phylogenetic analysis of the BoAstV strains. (**A**) Phylogenetic analysis of the BoAstV strains based on the whole-genome gene. (**B**) Phylogenetic analysis of the BoAstV strains based on the ORF2 gene. Note. The phylogenetic tree was generated based on the whole-genome gene and the ORF2 gene using the neighbor-joining method in MEGA 6.0 with 1000 bootstrap replicates. Strain name, symptom, year of collection, country, and the GenBank accession number are indicated.

Multiple genotypes of AstVs have been identified in other hosts, such as humans, bats and pigs [1]. Recent studies revealed that BoAstVs were composed of seven genotypes, namely, MAstV-13, MAstV 28–30 and MAstV 33–35, and the BoAstV strains were divided into five groups based on the complete genome nucleotide sequences [11]. In this study, the BoAstV reference strains (Table S1) were also divided into five groups based on the phylogenetic analysis of the full-length sequence. Most BoAstV strains from brain tissue were clustered into group 1 (G1), and strains from the intestinal tissue were clustered into group 3 (G3), group 4 (G4) and group 5 (G5). At present, only one respiratory BoAstV strain was identified as group 2 (G2), as seen in Figure 3A. Phylogenetic analysis of the ORF2 gene of global BoAstV strains also shows a similar classification as in Figure 3B. Further analysis shows that several species are closely related to the sequence classification of BoAstV. Based on phylogenetic analysis of the full-length sequence, the AstV strain from bovine (MK987099) is closely related to porcine (LC201603), while in brain tissue, the AstV strain from bovine (KT956903) is closely related to sheep (KY859988). In the ORF2 gene, the enteric AstV strain from bovine (LC047793), porcine (LC201620) and sheep (JN592482) are closely related.

## 5. Detection Methods

Electron microscopy (EM) has long been used for the detection and diagnosis of BoAstV [9] and RT-PCR methods have been developed for rapid detection [11]. Multiplex PCR assays are also being increasingly used for the diagnosis of calf viral diarrhea, including BoAstV [32]. Nested RT-PCR was used to detect BoAstV in the feces of healthy and diarrheic calves, and the RdRp gene was amplified [8] and TaqMan-based real-time PCR (qPCR) has also been used to detect and quantify BoAstV. This method has high sensitivity and specificity, and the real time method has a detection limit of 1.31 × 10^2^ RNA copies, which was 100-fold more sensitive than conventional PCR [33]. Random amplification and high-throughput sequencing are becoming more popular methods in discovering new viruses and solving macrogenome problems [2,19], while Italian researchers have established nanopore technology based on a macrogenome to more accurately detect astroviruses [25]. Boujon et al. (2017) developed an immunohistochemical (IHC) method to detect neurophilic BoAstV associated with bovine non suppurative encephalitis. Experiments confirmed that this method was an appropriate tool for detecting BoAstV infection [34]. Fluorescence in situ hybridization (ISH) was also used to detect BoAstV in bovine encephalitis cases [20].

## 6. Coinfection of BoAstV with Typical Bovine Enteric, Neurotropic and Respiratory Viruses

Some reports have shown that BoAstV is a potential pathogen causing enteric or neurotropic symptoms [2,9,35,36]. Although, it has been reported in the literature that BoAstV is controversial as to whether or not BoAstV causes clinical symptoms, the co-occurrence of this virus with circulating viruses has been reported since its discovery, as shown in Table 2. Among enteric pathogens, common co-infected viruses include bovine viral diarrhea virus (BVDV), bovine torovirus (BToV), bovine enteroviruses (BEV), bovine kobuvirus (BKV), bovine coronavirus (BcoV), bovine norovirus (BnoV), bovine rotavirus (BRV) and bovine enteric caliciviruses (BEC) [9,11,16]. Of these, BoAstV and BRV co-infection is reported most frequently, while the positivity rate of co-infection with BKV was the highest, at up to 66.67% [6,9,11]. Among the reports of co-infection involving three viruses, BoAstV with BRV and BnoV was the most common, with a positivity rate of 31.11% [16]. Among nervous system infectious pathogens, common co-infected viruses include parainfluenza virus 5 (PIV-5), bovine polyomavirus 2 (BpyV-2) and bovine herpesvirus 6 (BoHV-6), which cause bovine neurological symptoms [13,37]. In cases with respiratory symptoms, BoAstV was detected in the respiratory tract together with bovine adenovirus 3 (BadV-3), bovine rhinitis A virus (BRAV), bovine rhinitis B virus (BRBV) and influenza D virus (IDV) [27].

## 7. Pathogenesis of BoAstV

To date, the pathogenesis of BoAstV has not been determined and the detection of astroviruses in different tissues represents a wide range of tissue tropism. It is believed that, since BoAstV is highly prevalent in cattle populations and has an elevated level of genetic diversity, its pathogenesis may be determined by direct evidence of clinical disease. It was first detected in the intestines of calves with enteritis [9], in the brain tissue with neurological symptoms [6] and it was recently detected in nasal swab samples of respiratory diseases [27]. This shows that though BoAstV has been identified from different tissues of cattle, it is not clear which cells are responsible for its proliferation and transmission.

Previous reports showed that calves infected with BoAstV alone did not develop clinical disease, but when BoAstV was mixed with BRV or Breda virus 2, the calves developed severe diarrhea and more extensive astrovirus infection of the dome epithelium with associated degeneration [38,39]. Some reports showed that astrovirus may cause diarrhea in an unconventional way. Human astrovirus does not cause intestinal inflammation and cell death, but destroys tight junctions, leading to diarrhea [38]. Some studies suggested that the astrovirus capsid itself can cause diarrhea in vivo [40,41,42], and astrovirus infection alters mucus production, leading to an increase in mucus-associated bacteria [43].

With the discovery of bovine encephalitis cases, the association between astrovirus induced bovine nervous system diseases was also determined, but compared with these intestinal symptoms, the neurological symptoms have a greater impact. Selimovic-Hamza et al. (2016) suggested that astroviruses were already involved in the pathogenesis of European sporadic bovine encephalitis (ESBE) several decades ago, but had gone undetected. Researchers found astrovirus RNA in the brain of ESBE cases, but not in control animals, which is the basis of association with the disease. Studies have shown that astrovirus can exist in different brain regions, affecting not only neurons, but also inflammatory cells in perivascular cuffs and glial nodes [26]. Schlottau et al. (2016) detected a high level of BoAstV in the cervical spinal cord, cerebellar hemisphere and trigeminal ganglia of cows with neurological symptoms. Histopathology showed that acute encephalitis occurred in the brainstem and trigeminal ganglionitis and neuronal necrosis occurred in the brain and ganglia [20]. Using the combination of fluorescence in situ hybridization and immunofluorescence (IF) technology, it was possible to detect viral RNA and antigen in the same cell, indicating that the virus actively replicated in infected neurons [25]. In the case of human astrovirus associated encephalitis, humans with low immune function who are simultaneously affected by viral infection or management pressure may be more likely to develop nervous system diseases when infected with the astrovirus [44]. Currently, neurophilic BoAstV has not been isolated and cultured, which has affected the study of the pathogenesis of the disease.

In 2015, BoAstV was detected in nasal swab samples of bovines with respiratory diseases, but not in healthy cattle, which may indicate that it has potential respiratory pathogenicity. However, there was co-infection with other viruses in the BoAstV-positive nasal swab samples, so its pathogenicity has not been confirmed [27]. The pathogenesis of BoAstV infection may involve brain tissue, gastrointestinal tract and even respiratory tract [20,27,45], although there is evidence that astrovirus is transmitted through the fecal-oral route [44]. However, it is not clear whether there is an association between enteric, neurophilic and respiratory BoAstV and how the virus circulates in cattle. Studies have shown that neurophilic-astv (NT-AstV) may invade the central nervous system from the gastrointestinal tract retrograde along the peripheral nerves or the circulatory system, since there are cases in which NT-AstV infection induced dorsal root ganglionitis [19,20,46,47]. Recent reports also showed that neurophilic BoAstV was detected from bovine fecal samples, which also confirmed this possibility [48].

Other astroviruses are frequently detected in the gastrointestinal tract, which may be an important site of allergic infection and transmission and could be an important source of the virus to other body areas, leading to subsequent disease, however, the detection of BoAstV in nasal swabs of cattle suffering from a respiratory disease indicates a second site of virus entry. At present, one of the main obstacles to understanding the pathogenesis of astrovirus is the lack of a cell culture system suitable for most genotypes. Because this virus is difficult to culture in an immortalized stable cell line and usually coexists with other circulating bovine intestinal, neural and respiratory viruses, it is difficult to study the exact role of this virus in disease progression. Establishing a cell culture system suitable for most genotypes should be the focus for determining the pathogenic mechanism of BoAstV.

## 8. Cross Species Transmission of BoAstV

Astroviruses have a wide host range and high genetic diversity. It has been considered that astroviruses were species-specific; however, recently-discovered novel astroviruses and genetic and evolutionary studies of astroviruses show that they have the potential to cross species barriers and adapt to new host species [1,35,49,50]. The phylogenetic tree based on the complete genome sequence of BoAstV showed that bovine astrovirus (MK987099) and porcine astrovirus (LC201603) are located in the same branch, as in Figure 3A. In the evolution of the ORF2 gene, bovine (LC047793), sheep (JN592482) and porcine (LC201620) astroviruses are located in the same branch, bovine astrovirus (KT956903) and sheep astrovirus (KY859988) are also located in the same branch, as seen in Figure 3B.

At present, it is considered that the fecal–oral route may be the main route for the transmission of astrovirus. Feces or vomit, contaminated dust mites, transport and feed may be important sources of transmission. As AstV is a non-envelope virus, which can survive in harsh environments, studies have identified the presence of human astrovirus in groundwater and river water [51]. The long-term stability of AstV in water sources can provide a way to infect new hosts without requiring them to be physically close to each other [52]. Some reports showed that bacteria and bacterial components such as lipopolysaccharides can maintain the infectivity of human astroviruses by stabilizing the capsid, allowing continuing infectivity for a long time after abscission, which may also have a far-reaching impact on the transmission of astrovirus between hosts [53]. Recombination may be another key factor for cross species transmission of the virus and has been reported in human and sea lion astroviruses [41,54]. Tse et al. (2011) reported co-infection of different astrovirus genotypes in one cow, which is also a necessary step for recombination [14]. At present, there is no evidence that BoAstV is associated with human astrovirus, but the characteristics of frequent close contact and frequent recombination of the viral genome may provide potential conditions for cross species transmission, which deserves further study.

## 9. Conclusions and Future Prospects for BoAstV

BoAstV is prevalent in many countries around the world. The reports of BoAstV in cattle with intestinal disease, neurological symptoms and even respiratory disease indicates that it has multiple tissue tropism and may cause serious economic losses for the global cattle industry. Human, chicken and murine astrovirus researchers have made progress in cell culture and pathogenesis [55,56,57,58], but the difficulty of isolation and culture limits further research on BoAstV [14]. For a variety of problems and future research directions of BoAstV, it is worth discussing the following aspects:

### 9.1. Establishment of a BoAstV Culture System

At present, BoAstV is only reported to be cultured in several primary cells, but the isolation and culture of neurogenic and respiratory BoAstV has not been reported. The difficulty of stabilizing cell line culture limits further research on BoAstV. In humans, HAstV have been replicated in a variety of cell lines, among which, human colon cancer cell type 2 (Caco-2) is the most widely used. Several cell lines have been trialed to isolate BoAstV [14], but a stable passage cell line for isolating BoAstV has not been reported. The identification of cell receptors is also needed to help understand the proliferation and culture of viruses in cells, which requires the determination of BoAstV cell receptors as soon as possible.

The development of 3D enteroid cells has immense potential as a model to explore and better understand BoAstV. Protocols for developing 3D enteroid cells already exist for several species infected by AstVs, including pigs, chickens and bats [55,59,60]. Using an epithelium-only human intestinal enteroid (HIE) system, Kolawole et al. (2019) identified for the first time that HAstV infects several cell types, including intestinal progenitor cells and mature enterocytes [57]. These findings may provide future reference and insight for future studies on BoAstV.

### 9.2. Potential Tropism of Other Organs

BoAstV has been reported in intestines, brain tissues and the respiratory tract. In many animals, astroviruses are also found in other organs. Duck astrovirus can cause duck hepatitis, goose astrovirus, chicken astrovirus and Avian nephritis virus can cause kidney disease [56,61,62]. In addition, astrovirus RNA has been detected in infected human hearts [63]. Histological results of myocarditis and epicarditis were identified from two pigs infected with porcine astrovirus [64]. It is unclear whether BoAstV can infect cells of the immune or circulatory system, whether it has tissue tropism to the liver, kidney and other organs, and whether virus infection will cause systemic diseases has not been reported. These potential tissue trends highlight the future direction for research, which can only be further evaluated by an in vivo model of astrovirus infection.

### 9.3. Pathogenic Significance of BoAstV

Although BoAstV has been found for decades, it has not received the same attention as other viral RNA families. This lack of interest is partly due to the assumption that astroviruses only cause self-limiting gastrointestinal diseases in humans and other vertebrates [9]. However, studies have found that BoAstV may be involved in the co pathogenic effects of other bovine enteroviruses [65] and some reports show that BoAstV may play a more significant role in nervous system diseases. BoAstV has been detected in the brain tissue of many cases of encephalitis [2,26]. Phylogenetic analysis showed that BoAstV is closely related to the neurophilic astrovirus of other species, and it is speculated that they may have similar pathogenic mechanisms and the potential for cross-species transmission. The study of BoAstV is of great significance not only for the healthy breeding of cattle, but also for the study of nervous system injury and disease transmission of astrovirus in other animals and even humans.

### 9.4. Exploration and Future Direction of Astrovirus Infection Spectrum

Astroviruses infect a wide range of hosts, and have been detected in over 80 avian and mammalian host species to date, affecting both public health systems and economic production chains [1]. According to literature data, the presence of ecotones and frequent recombination of intra-species and inter-species are likely to be key factors for cross species transmission of different host astroviruses, including BoAstV [50]. However, the exact cross species transmission mechanisms have not been determined. It is inevitable that many more different host astroviruses are likely to be discovered and more sequences determined, given the enormous advances in metagenomic and next-generation sequencing technologies. This would help the understanding of the ecological drivers that facilitate the evolution, amplification and dampening of astroviruses across interfaces. Although the receptor of astroviruses has not yet been determined, it has been shown that astroviruses infect cells through capsid protein-mediated binding to cell surface receptors. The comparative analysis of crystal structure and morphology of capsid spike domains as the primary binding site in different hosts may be beneficial to the study of potential cross species transmission between different hosts.

## Figures and Tables

**Table 2:** General information of documented BoAstV cases.

Order	Year	Country	Sample Type	Co-Infection	References
1	1978	United Kingdom	stool samples/rectal swab	BEC, BRV	[9]
2	2014	Korea	stool samples/rectal swab	BcoV, BVDV, BKV, BRV	[6]
3	2015	China	stool samples/rectal swab	BtoV, BVDV, BEV, BcoV BRV	[16]
4	2016	Switzerland	brain tissue samples	BoHV-6	[13]
5	2017	Egypt	stool samples/rectal swab	BnoV, BRV	[5]
6	2021	China	stool samples/rectal swab	BRV, BKV, BEV	[11]
7	2016	Switzerland	brain tissue samples	BoHV-6	[13]
8	2020	Switzerland	brain tissue samples	PIV-5, BpyV-2, BoHV6	[37]
9	2015	USA	nasal swabs samples	BadV-3, BRAV, BRBV, IDV	[27]

## Data Availability

Data sharing not applicable.

## Supplementary Materials

The following supporting information can be downloaded at: https://www.mdpi.com/article/10.3390/v14061217/s1, Table S1: The reference AstV strains used in this study.
