# Supplementary material for: Bovine Astrovirus—A Comprehensive Review
Source: Viruses. 2022 Jun 2;14(6):1217. doi: 10.3390/v14061217 (PMC9228355; doi:10.3390/v14061217)
Supplement: Supplementary file 1 [file viruses-14-01217-s001.zip › viruses-1687656-supplementary.pdf]

Table S1. The reference AstV strains used in this study.

Strains name	Hosts	GenBank accession no.	Symptom	Collection date	Countries
B18/HK	Bovine	HQ916313	Normal	2011	China
B170/HK	Bovine	HQ916314	Normal	2011	China
B34/HK	Bovine	HQ916315	Normal	2011	China
BoAstV-GX-J27	Bovine	KJ476832	Diarrhea	2013	China
BoAstV-GX-G1	Bovine	KJ476833	Diarrhea	2013	China
BoAstV-GX-J7	Bovine	KJ476834	Diarrhea	2013	China
BoAstV-GX-J22	Bovine	KJ476835	Diarrhea	2013	China
BoAstV-GX7/CHN/2014	Bovine	KJ620979	Diarrhea	2014	China
HMO-CLondon2	Human	KJ920197	Normal	2010	USA
Takin astrovirus	Sichuan takin	NC_037655	Normal	2013	China
DA06	Duck	FJ919225	Unknown	2016	China
CHN/2017/44	Canis lupus familiaris	MF973500	Diarrhea	2017	China
BoAstV/JPN/Hokkaido11-7/2009	Bovine	LC047789	Diarrhea	2009	Japan
BoAstV/JPN/Hokkaido11-55/2009	Bovine	LC047790	Diarrhea	2009	Japan
BoAstV/JPN/Hokkaido12-7/2009	Bovine	LC047791	Diarrhea	2009	Japan
BoAstV/JPN/Hokkaido12-18/2009	Bovine	LC047792	Diarrhea	2009	Japan
BoAstV/JPN/Hokkaido12-25/2009	Bovine	LC047793	Diarrhea	2009	Japan
BoAstV/JPN/Hokkaido12-27	Bovine	LC047794	Diarrhea	2009	Japan
BoAstV/JPN/Ishikawa24-6/2013	Bovine	LC047787	Normal	2013	Japan
BoAstV/JPN/Ishikawa9728/2013	Bovine	LC047788	Normal	2013	Japan
BoAstV/JPN/Kagoshima1-2/2014	Bovine	LC047795	Normal	2014	Japan
BoAstV/JPN/Kagoshima1-7/2014	Bovine	LC047796	Diarrhea	2014	Japan

BoAstV/JPN/Kagoshima2-3-1/2015	Bovine	LC047797	Diarrhea	2015	Japan
BoAstV/JPN/Kagoshima2-3-2/2015	Bovine	LC047798	Diarrhea	2015	Japan
BoAstV/JPN/Kagoshima2-24/2015	Bovine	LC047799	Diarrhea	2015	Japan
BoAstV/JPN/Kagoshima2-52/2015	Bovine	LC047801	Diarrhea	2015	Japan
BoAstV/JPN/Kagoshima2-38/2015	Bovine	LC047800	Diarrhea	2015	Japan
BoAstV/JPN/KagoshimaSR28-462/2016	Bovine	LC341267	Diarrhea	2016	Japan
PoAstV-2/JPN/Bu5-10-1/2014	Porcine	LC201585	Normal	2014	Japan
PoAstV-3/JPN/Bu2-5/2014	Porcine	LC201595	Normal	2014	Japan
PoAstV-4/JPN/Bu5-10-2/2014	Porcine	LC201603	Normal	2014	Japan
PoAstV-5/JPN/Ishi-Im1-2/2015	Porcine	LC201620	Normal	2015	Japan
Bovine astrovirus NeuroS1	Bovine	KF233994	Encephalitis	2011	USA
BSRI-1	Bovine	KP264970	Respiratory disease	2013	USA
9715	Sea lion	JN420358	Normal	2010	USA
FAstV-D1	Feline	KM017741	Unknown	2013	USA
MAstV/Bov/ITA/2012/715	Bovine	KT963071	Diarrhea	2012	Italy
BoAstV-Neuro-Uy	Bovine	MK386569	Encephalitis	2018	Uruguay
LVMS2704	Bovine	MN200263	Unknown	2016	Uruguay
CH13	Bovine	KM035759	Encephalitis	2012	Switzerland
Bovine astrovirus CH13/NeuroS1 isolate 26875	Bovine	KX266903	Encephalitis	2015	Switzerland
Bovine astrovirus CH13/NeuroS1 isolate 36716	Bovine	KX266904	Encephalitis	2015	Switzerland
Bovine astrovirus CH13/NeuroS1 isolate 23985	Bovine	KX266905	Encephalitis	2015	Switzerland
Bovine astrovirus CH13/NeuroS1	Bovine	KX266907	Encephalitis	2015	Switzerland

isolate 43661					
Bovine astrovirus CH13/NeuroS1 isolate 43660	Bovine	KX266908	Encephalitis	2015	Switzerland
Bovine astrovirus CH13/NeuroS1 isolate 26730	Bovine	KX266902	Encephalitis	2015	Switzerland
Bovine astrovirus CH13/NeuroS1 isolate 42799	Bovine	KX266906	Encephalitis	2015	Switzerland
Bovine astrovirus CH13/NeuroS1 isolate 23871	Bovine	KX266906	Encephalitis	2015	Switzerland
CH15	Bovine	KT956903	Unknown	2015	Switzerland
BoAstV-VC34/338	Bovine	MK987099	Encephalitis	2016	Switzerland
BoAstV-VC34/346	Bovine	MK987100	Encephalitis	2016	Switzerland
BoAstV-VC65/698	Bovine	MK987103	Encephalitis	2016	Switzerland
BoAstV-VC34/375	Bovine	MK987101	Encephalitis	2016	Switzerland
BoAstV-VC65/693	Bovine	MK987102	Encephalitis	2016	Switzerland
MOxAstV-CH18	Ovibos	MK211323	Unknown	1982	Switzerland
CH16	Sheep	KY859988	Encephalitis	2006	Switzerland
CcAstV/roe_deer/SLO/D5-14/2014	Roe deer	MN150124	Normal	2014	Slovenia
CcAstV/roe_deer/SLO/D12-14/2014	Roe deer	MN150125	Normal	2014	Slovenia
OAstV-2/Hungary/2009	Sheep	JN592482	Normal	2009	Hungary
Hu/Nyergesujfalu/HUN4520/2010/HUN	Human	HQ398856	Diarrhea	2010	Hungary
UK/2013/ewe/lib01454	Sheep	LT706531	Unknown	2013	UK
UK/2014/lamb/lib01454	Sheep	LT706530	Unknown	2014	UK
ANV-2-VF07-13-7_UK_2007	Avian	HQ330482	Unknown	2007	UK
BH89/14	Bovine	LN879482	Encephalitis	2014	Germany
BtnMLB1-40	Human	AB823731	Diarrhea	2010	Bhutan

DcAstV-274	Camelus	KR868724	Unknown	2013	United Arab Emirates
Bat-AsV/P02	Bat	MG693176	Normal	2013	Cameroon
Sheep astrovirus	Sheep	Y15937	Unknown	1997	Norway
Mink astrovirus	Mink	AY179509	Normal	2002	Unknown
BAstV-GX27/CHN/2014	Bovine	KJ620980	Diarrhea	2014	China
LVMS681	Bovine	MN200262	Unknown	2015	Uruguay
